# Prolonged Chronic Consumption of a High Fat with Sucrose Diet Alters the Morphology of the Small Intestine

**DOI:** 10.3390/ijms22147280

**Published:** 2021-07-06

**Authors:** Roberta Sferra, Simona Pompili, Alfredo Cappariello, Eugenio Gaudio, Giovanni Latella, Antonella Vetuschi

**Affiliations:** 1 Department of Biotechnological and Applied Sciences, University of L’Aquila, 67100 L’Aquila, Italy; simona.pompili@guest.univaq.it (S.P.); alfredo.cappariello@guest.univaq.it (A.C.); antonella.vetuschi@univaq.it (A.V.); 2 Department of Anatomical, Histological, Forensic Medicine and Orthopedic Sciences, Sapienza University of Rome, 00161 Rome, Italy; eugenio.gaudio@uniroma1.it; 3 Department of Life, Health and Environmental Sciences, Division of Gastroenterology, Hepatology, and Nutrition, University of L’Aquila, 67100 L’Aquila, Italy; giolatel@tin.it

**Keywords:** intestine, diet, lipids, lipid droplets, fat, intestinal inflammation, small bowel

## Abstract

(1) The high-fat diet (HFD) of western countries has dramatic effect on the health of several organs, including the digestive tract, leading to the accumulation of fats that can also trigger a chronic inflammatory process, such as that which occurs in non-alcohol steatohepatitis. The effects of a HFD on the small intestine, the organ involved in the absorption of this class of nutrients, are still poorly investigated. (2) To address this aspect, we administered a combined HFD with sucrose (HFD w/Suc, fat: 58% Kcal) regimen (18 months) to mice and investigated the morphological and molecular changes that occurred in the wall of proximal tract of the small intestine compared to the intestine of mice fed with a standard diet (SD) (fat: 18% Kcal). (3) We found an accumulation of lipid droplets in the mucosa of HFD w/Suc-fed mice that led to a disarrangement of mucosa architecture. Furthermore, we assessed the expression of several key players involved in lipid metabolism and inflammation, such as perilipin, leptin, leptin receptor, PI3K, p-mTOR, p-Akt, and TNF-α. All these molecules were increased in HFD mice compared to the SD group. We also evaluated anti-inflammatory molecules like adiponectin, adiponectin receptor, and PPAR-γ, and observed their significant reduction in the HFD w/Suc group compared to the control. Our data are in line with the knowledge that improper eating habits present a primary harmful assault on the bowel and the entire body’s health. (4) These results represent a promising starting point for future studies, helping to better understand the complex and not fully elucidated spectrum of intestinal alterations induced by the overconsumption of fat.

## 1. Introduction

The small intestine is actively involved in the metabolism of dietary fats, such as cholesteryl esters (CEs), phospholipids (PLs), and triglycerides (TGs); among these, approximately 90% of the total fat calories are provided by TGs [1,2,3,4]. The first step in lipid digestion and emulsification occurs in the mouth and stomach due to the combined action of salivary and gastric lipases. Pancreatic lipases and bile acids then cooperate to further digest and emulsify lipids, facilitating their absorption in the small intestine. Lipases split TGs into fatty acids and monoglycerides, which are packaged in mixed micelles, making lipids absorbable by the intestinal epithelium [1,2,3,4]. Once inside the small intestine, they are resynthesized, and fatty acids and monoglycerides reach the endoplasmic reticulum membrane, where they have two fates: a portion of them get into the endoplasmic reticulum and are transported into the Golgi apparatus and secreted into the bloodstream as chylomicrons [4,5,6,7]. The other portion of lipids are stored in cytosolic lipid droplets (CLDs), which are spherical particles surrounded by a phospholipid monolayer and covered on the surface by several proteins, mainly members of the perilipin family. In physiological conditions, CLDs are temporarily accumulated in the cytosol of enterocytes as an energy reservoir and are released for energy supply during the intestinal fasting state [4,5,6,7]. However, when excessive accumulation of lipids occurs, as the result of an unbalanced diet or alterations in lipogenesis/lipolysis processes, severe diseases may result [8,9,10,11,12,13,14,15,16,17,18].

A surplus of TGs induces liver injury, such as nonalcoholic fatty liver disease (NAFLD), nonalcoholic steatohepatitis (NASH), and even cirrhosis and hepatocarcinoma [12,16,17,18]. They may also be responsible for pancreatic damage by reducing apoptosis of β-cells, with the consequent reduction in insulin secretion [14,15]. Moreover, the accumulation of TGs in skeletal muscle leads to insulin resistance and a reduction of glucose uptake [10,19], while accumulation in the myocardium results in cardiomyopathy [9,11].

Although the intestinal tract is known to be directly involved in lipid metabolism, the mucosal alterations induced by an enriched fat diet are still limited [20,21].

Indeed, several studies have focused attention on the changes in intestinal microbiota and the exacerbation of several intestinal disorders (i.e., food allergies, inflammatory bowel disease (IBD), or colorectal cancer) [22,23,24,25], while a few studies have evaluated the impact of a high-fat diet (HFD) on intestinal inflammation [26,27,28,29]. 

Lipid metabolism is regulated by several molecules, including peroxisome proliferator-activated receptor-γ (PPAR-γ), a master gene of adipogenesis and adipocyte differentiation that is also involved in the regulation of many processes, such as cell proliferation, differentiation, inflammation, and fibrosis [30,31,32]; for this reason, PPAR-γ could be a promising therapeutic target. It has been demonstrated that rosiglitazone and other molecules that are able to enhance PPAR-γ functions lead to an improvement in inflammation and metabolic alterations induced by excessive fat intake [30,31,32]. 

A HFD also increases the expression of adipokines involved in lipid metabolism, such as leptin, which are responsible for a downregulation of PPAR-γ and the upregulation of proinflammatory cytokines like TNF-α and IL-6 through the PI3K/mTOR pathway [33,34,35,36].

In previous studies, we demonstrated that the abuse of an 18-month long chronic hyperlipidemic diet induced severe metabolic dysfunctions altering the mice’s body weight, liver weight/body weight ratio, serum parameters like ALT, AST, triglycerides, and cholesterol and prompted the liver parenchyma towards nonalcoholic steatohepatitis (NASH) and hepatocarcinoma [17,18]. On this basis, we hypothesized that the same dietary regimen may directly induce also alterations of bowel wall morphology and that the accumulation of fat in the mucosa may induce mucosal damage and may trigger the onset of intestinal inflammation. This study aimed to make a pairwise evaluation of the impact of the chronic administration of an HFD w/Suc on the small intestine of the same mice already evaluated for liver phenotype in our previous studies [17,18]. 

## 2. Results

### 2.1. Morphological Changes in the Small Intestine Architecture Induced by a High Fat with Sucrose Diet

**Figure 1:**
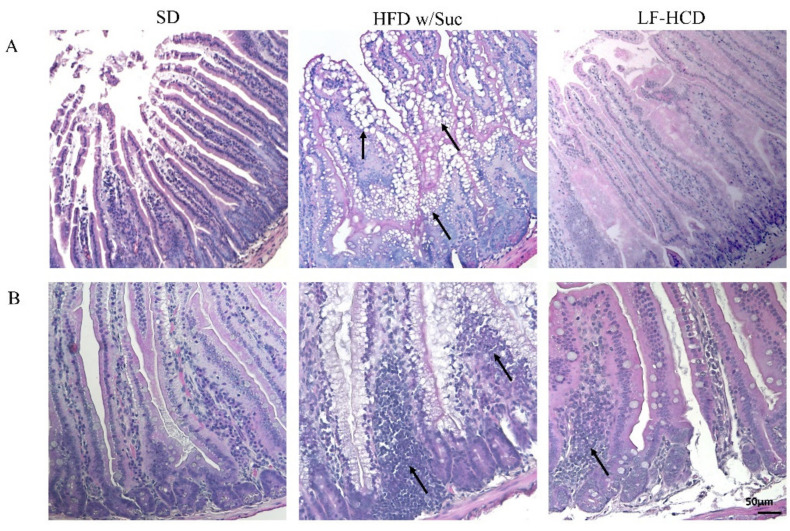
Hematoxylin and Eosin staining of proximal tract of the small intestine. Original magnification: 20×; scale bar: 50 μm. (**A**) A marked accumulation of lipid droplets was present in the intestinal mucosa of HFD w/Suc -fed mice (arrows) compared to SD-fed and LF-HCD-fed mice. (**B**) A massive infiltration of inflammatory cells was found in HFD w/Suc-fed mice compared to the SD group, while LF-HCD-fed mice showed only scanty and mild inflammatory foci (arrows). These images are representative of *n* = 5 SD, *n* = 10 HFD w/Suc, *n* = 10 LF-HCD mice.

Histological analyses by H&E staining showed a marked accumulation of lipid droplets in the mucosa of HFD w/Suc-fed mice, leading to a disruption of normal intestinal architecture (Figure 1A). 

Additionally, in the HFD w/Suc mice, a spread inflammatory infiltrate was found across the mucosa (Figure 1B), whereas no signs of CLD deposition and relevant inflammatory foci were detected in the SD group (Figure 1A,B). Interestingly, the analysis of the small intestine from mice fed with a hypercaloric low-fat (4.8%), high-sucrose (61.12%) diet did not show significant signs of tissue alterations and lipidic accumulation or signs of significant inflammation. These findings suggest that the lipidic content of the diet can specifically induce intestinal alterations.

**Figure 2:**
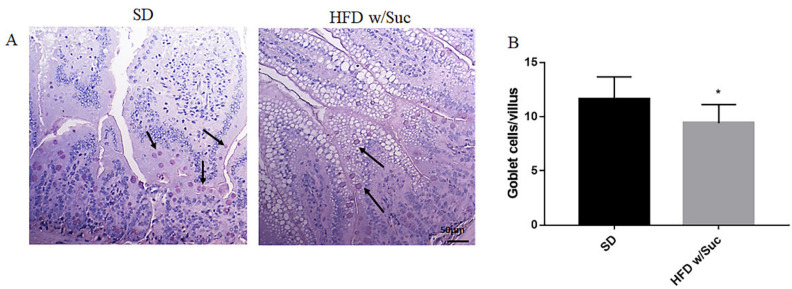
(**A**) PAS staining of proximal tract of the small intestine. Original magnification: 20×; scale bar: 50 μm. (**B**) Quantification of goblet cells. HFD w/Suc mice showed a reduction of goblet cell numbers compared to the SD group (arrows). * *p* < 0.05. These images are representative of *n* = 5 SD, *n* = 10 HFD w/Suc mice.

To highlight the dietary effects on intestinal epithelial barrier components, PAS staining elective for goblet cells was also undertaken (Figure 2A). The goblet cells secrete numerous mucins that form a thin layer of mucus adhering to the mucosal surface, which represents an important component of the intestinal mucosal barrier. The quantitative evaluation revealed a significant reduction of goblet cells in HFD w/Suc mice compared to SD mice (Figure 2B).

**Figure 3:**
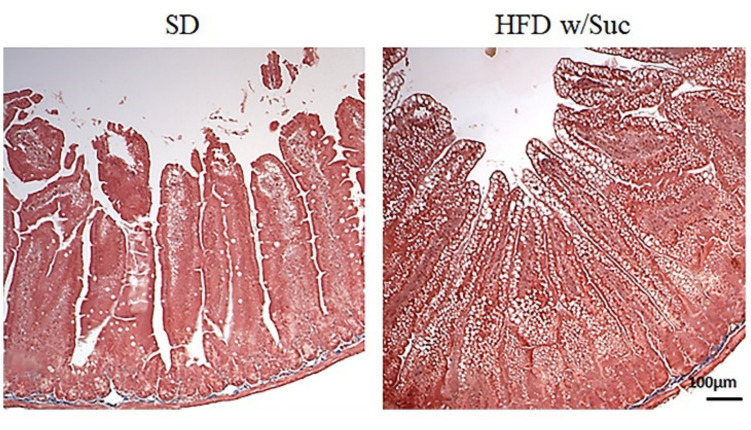
Masson’s trichrome staining of proximal tract of the small intestine. (Original magnification: 10×; scale bar: 100 μm). No signs of fibrosis were found in either the SD or the HFD w/Suc -fed mice. These images are representative of *n* = 5 SD, *n* = 10 HFD w/Suc mice.

Fibrosis was absent in both the SD and HFD w/Suc mice (Figure 3).

### 2.2. Evaluation of Protein Changes Induced by a High-Fat with Sucrose Diet

**Figure 4:**
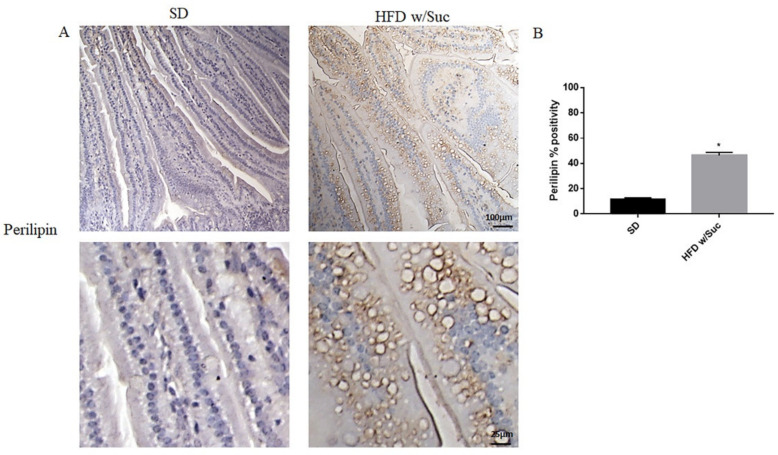
(**A**) Immunohistochemistry for perilipin 1 of proximal tract of the small intestine. (Upper: original magnification: 10×; scale bar: 100 μm, bottom: magnification 40×; scale bar: 25 μm). (**B**) The expression of perilipin 1 was significantly increased in HFD w/Suc mice compared to the SD group. * *p* <0.05. These images are representative of *n* = 5 SD, *n* = 10 HFD w/Suc mice.

To confirm the presence of lipids, we performed immunohistochemistry analysis for perilipin 1, which is one of the CDL surface proteins (Figure 4A).

HFD w/Suc mice showed increased perilipin expression compared to the control group, mainly localized on the rim of the lipid droplets present in the intestinal mucosa layer (Figure 4A). The increase of perilipin in the HDF w/Suc group was confirmed by semi-quantitative analysis (Figure 4B).

**Figure 5:**
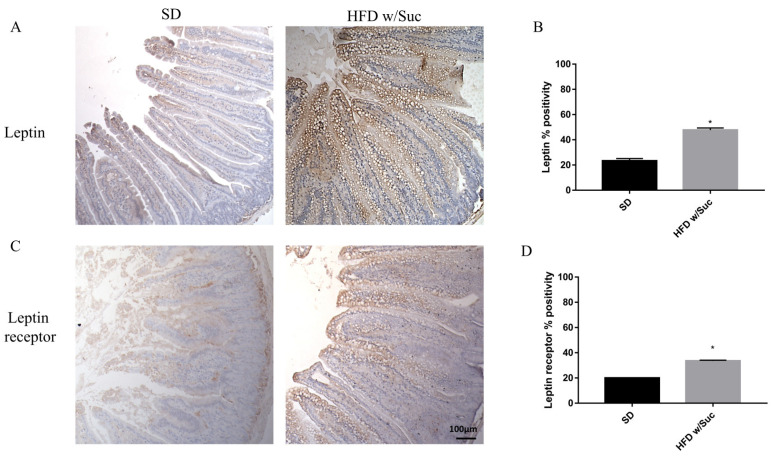
(**A**,**C**) Immunohistochemistry for leptin and leptin receptor of proximal tract of the small intestine. (Original magnification: 10×; scale bar: 100 μm). Immunostaining of both leptin and its receptor showed an increase in HFD w/Suc mice compared to the SD group. (**B**,**D**) Semiquantitative analyses of leptin and leptin receptors confirmed the increased expression of these two molecules. * *p* < 0.05. These images are representative of *n* = 5 SD, *n* = 10 HFD w/Suc mice.

To confirm that a HFD w/Suc leads to an overexpression of leptin, we assessed this molecule and its receptor (Figure 5).

Immunostaining of leptin is noticeably increased in HFD w/Suc-fed mice compared to the SD group (Figure 5A,B). Evaluation of leptin receptor also showed significant differences between SD and HFD w/Suc-fed mice (Figure 5C,D).

**Figure 6:**
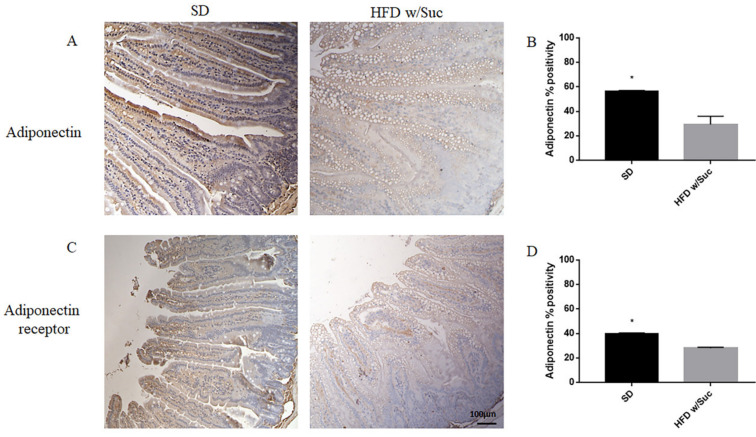
(**A**,**C**) Immunohistochemistry of adiponectin and adiponectin receptor of proximal tract of the small intestine. (Original magnification: 10×; scale bar: 100 μm). (**B**,**D**) The expression of adiponectin and its receptor was significantly reduced in HFD w/Suc mice compared to the SD group. * *p* < 0.05. These images are representative of *n* = 5 SD, *n* = 10 HFD w/Suc mice.

Furthermore, we performed an immunohistochemistry evaluation of adiponectin, a molecule that generally counterbalances the activity of leptin (Figure 6A).

The analysis revealed a significant reduction of adiponectin expression in the HFD w/Suc group compared to control mice (Figure 6A), as validated by the semiquantitative analysis (Figure 6B).

Similarly, adiponectin receptor immunopositivity was reduced in the HFD w/Suc group compared to the SD mice (Figure 6C,D).

**Figure 7:**
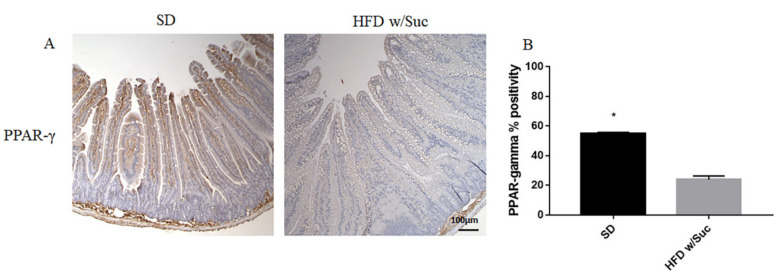
(**A**) Immunohistochemistry of PPAR-γ of proximal tract of the small intestine. (Original magnification: 10×; scale bar: 100 μm). (**B**) PPAR-γ was significantly reduced in HFD w/Suc mice compared to the SD group. * *p* < 0.05. These images are representative of *n* = 5 SD, *n* = 10 HFD w/Suc mice.

Immunohistochemistry of PPAR-γ showed a reduction of positivity in HFD w/Suc -fed mice compared to the SD group (Figure 7A), as confirmed by the semiquantitative analysis (Figure 7B). These data are in line with the anti-inflammatory role of this transcriptional factor [32]. 

**Figure 8:**
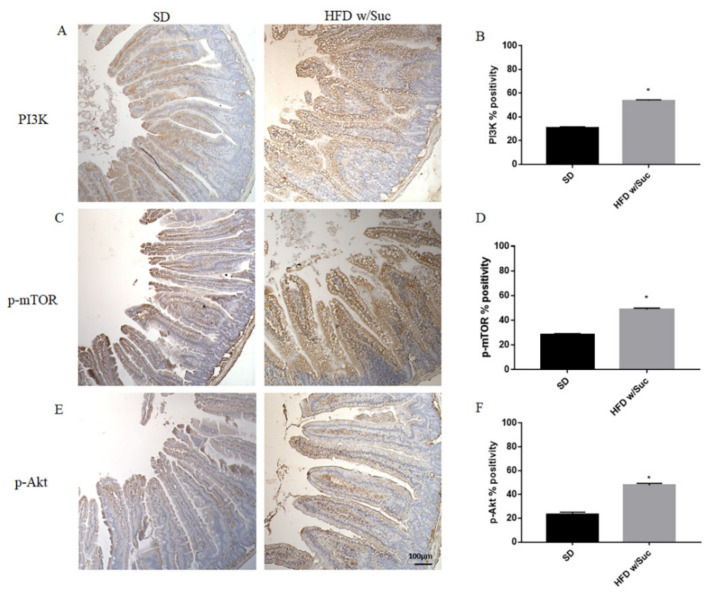
(**A**,**C**,**E**) Immunohistochemistry of PI3K, p-mTOR, and p-Akt of proximal tract of the small intestine. (Original magnification: 10×; scale bar: 100 μm). (**B**,**D**,**F**) All the tested molecules were increased in the HFD w/Suc group compared to SD mice. * *p* < 0.05. These images are representative of *n* = 5 SD, *n* = 10 HFD w/Suc mice.

Since the increase of lipid droplets was associated with a concomitant increase of mucosal inflammatory infiltrate in HDF w/Suc mice, we performed immunohistochemistry analysis for the proinflammatory mediators PI3K, phosphorylated-mTOR (p-mTOR), and phosphorylated Akt (p-Akt) (Figure 8) [37,38,39].

**Figure 9:**
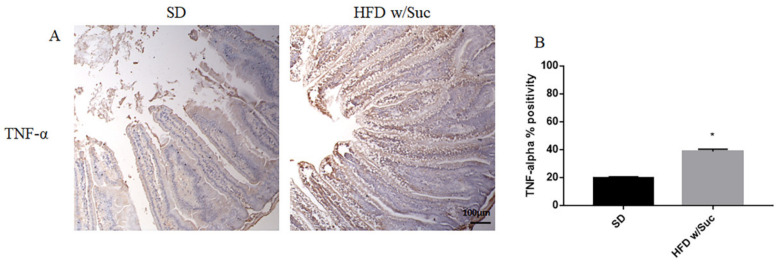
(**A**) Immunohistochemistry of TNF-α of proximal tract of the small intestine. (Original magnification: 10×; scale bar: 100 μm). (**B**) TNF-α showed an increase in the HFD w/Suc group compared to SD mice. * *p* < 0.05. These images are representative of *n* = 5 SD, *n* = 10 HFD w/Suc mice.

Immunostaining of PI3K, p-mTOR, and p-Akt was increased in the HFD w/Suc group compared to the SD-fed mice (Figure 8A,C,E), which was supported by the semiquantitative analyses of each of these examined molecules (Figure 8B,D,F). In addition, we evaluated the expression of TNF-α, one of the main proinflammatory factors (Figure 9).

Immunohistochemistry (Figure 9A) and semiquantitative evaluation (Figure 9B) revealed a significant increase of TNF-α in HFD w/Suc-fed mice compared to the SD group, confirming the onset of an inflammatory process after the consumption of a hyperlipidic diet.

**Figure 10:**
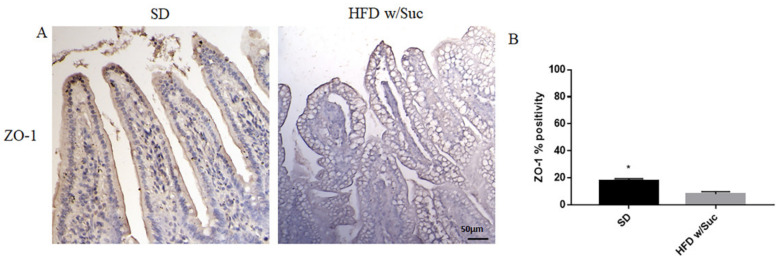
(**A**) Immunohistochemistry for Zonulin 1 (ZO-1) of proximal tract of the small intestine. (Original magnification: 20×; scale bar: 50 μm). (**B**) ZO-1 showed an increase in the SD group compared to HFD w/Suc mice. These images are representative of *n* = 5 SD, *n* = 10 HFD w/Suc mice.

Finally, to investigate the integrity of the intestinal epithelium, we evaluated the expression of Zonulin-1, a key molecule in the formation and remodeling of tight junctions and the maintenance of the intestinal barrier. Immunohistochemical analysis showed a marked decrease in the expression of zonulin-1 in the epithelium of the group of mice receiving HFD w/Suc compared to control mice (Figure 10), supporting the hypothesis that the hyperlipidic diet may alter the functions of the intestinal epithelial barrier.

## 3. Discussion

Dietary lipids are absorbed by the small intestine and, after their resynthesis, are transported to the endoplasmic reticulum where they are packaged in chylomicrons or temporarily stored in enterocytes in the form of CLDs as an energy reserve [5]. However, impairment in TG secretion due to an exaggerated lipid intake leads to the accumulation of CLDs and, consequently, to organ dysfunction. Over the last few decades, excessive consumption of a western-style diet, rich in fat and lower in vegetables and fibers, is increasingly becoming one of the main risk factors for several metabolic and chronic inflammatory disorders involving several organs. Indeed, besides obesity and metabolic syndrome, many diseases that affect a wide range of organs, such as NAFLD/NASH, human immunodeficiency virus (HIV)/simian immunodeficiency virus (SIV), cardiovascular disease, and Alzheimer’s disease, are induced or worsened by a chronic HFD regimen [17,18,40,41,42]. Furthermore, a direct correlation between excessive fat intake and the development/progression of various enteropathies (i.e., IBD, celiac disease, and irritable bowel syndrome), alteration of the intestinal microbiota, dysfunction of the epithelial barrier, and reduction of intestinal permeability has been reported [23,24,43]. However, there are limited data regarding the impact that an excessive intake of lipids has on the morphology of the intestinal mucosa and the onset of intestinal inflammation [27,28,44]. 

Lipid metabolism, as well as inflammation, are orchestrated by several molecules, including the adipokines leptin and adiponectin, which generally influence cellular behavior, acting in an opposite manner. In the physiological condition, leptin has a dual role, regulating food intake and body weight and inducing an inflammatory response, an action that is counterbalanced by adiponectin [35]. However, metabolic alterations are associated with an overexpression of leptin, as occurs after a hyperlipidic diet. In obese individuals, elevated levels of this molecule represent a predisposing factor to low-grade inflammation and contribute to the development of metabolic-syndrome-related diseases [35]. This condition can fuel a wider worsening of the whole organism affecting many systems, mainly cardiovascular and musculoskeletal, since they share common inflammatory members, such as IL-6 [44,45,46,47,48].

In this study we administrated a Surwit diet designed for diet-induced obesity studies, characterized by a combination of a high fat (58% kcal) and sucrose (12.59% kcal) content [49]. This diet was selected in order to guarantee a prolonged regimen, being a mono-nutrient fat enriched diet not suitable for palatability and metabolic (i.e., ketoacidosis) issues. The composition is consistent with a very high-fat content (60% kcal of fats), accordingly with general guidelines for research diet [50]. 

We demonstrate that the prolonged (up to 18 months) administration of a HFD w/Suc, leading to an accumulation of CLDs in the intestinal mucosa of the mice, is associated with relevant changes in the organ architecture, characterized by alterations of the typical villus conformation and increased inflammatory infiltrate in the lamina propria. Similar CLDs accumulation was reported in the intestine of animals but in different experimental conditions [51,52]. Interestingly, the intestinal phenotype of high-carbohydrates (sucrose enriched, 60.10% kcal)/low-fat (10.5% kcal) diet-fed mice was comparable to that of the control mice, providing evidence for HFD w/Suc -dependent mucosal alteration. These histological modifications of the intestinal mucosa were accompanied by a reduction in goblet cell numbers in HFD w/Suc-mice, in line with other previous studies [53,54]. The goblet cells release numerous mucins that contribute to the formation of the thin layer of mucus adhering to the epithelial surface, which acts as an important component of the intestinal mucosa barrier.

CLDs are characterized by a core of Triglycerides (TAGs) and cholesterol esters surrounded by a monolayer of PLs, cholesterol, and several proteins, including members of the perilipin family. 

Accordingly, the immunohistochemistry of perilipin highlighted a visible positivity in the rim of the lipid droplets in HFD w/Suc-fed mice, confirming the presence of adipose-like cells. 

**Figure 11:**
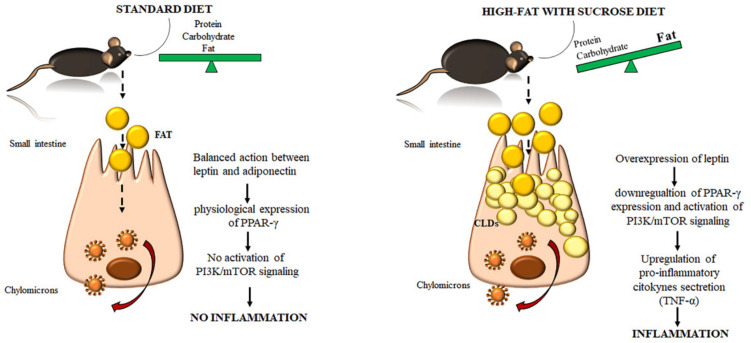
Cartoon showing the accumulation of cytoplasmatic lipid droplets (CDLs) and the onset of the inflammatory process induced by the consumption of an unbalanced diet. In a standard diet (SD) regimen, the fats are absorbed by enterocytes, processed, and sorted in chylomicrons upon the orchestrated action of leptin/PPAR-γ signaling. Conversely, the administration of a high-fat with sucrose diet (HFD w/Suc) induces an accumulation of CLDs in the small intestine of mice. Consequently, high levels of leptin lead to a downregulation of the anti-inflammatory molecule (PPAR-γ). Contextually, there was activation of the proinflammatory signaling driven by the PI3K/mTOR pathway and TNF-α, leading to mucosal inflammation.

We found a significant increase in leptin expression and a reduction of adiponectin in the HFD w/Suc group. Conversely, PPAR-γ positivity was more evident in SD mice compared to the HFD w/Suc group, where there was only a slight expression. PI3K, p-mTOR, and p-Akt, as well as TNF-α, were markedly increased in HFD-fed w/Suc mice compared to control mice receiving a SD, suggesting their role in the onset of the inflammatory process (Figure 11).

Since similar alterations in the expression of adipokines, PPAR-γ, PI3K, p-mTOR, p-Akt, and TNF-α have been reported in experimental colitis and IBD, a hyperlipidic diet could have adverse effects on the course of IBD [55,56,57,58,59,60]. If these data are confirmed in clinical studies, the hyperlipidic unbalanced diet should be avoided in enteropathies and in particular in IBD [61,62].

There is evidence that the western, high-caloric and HFD influences IBD in patients and rodent models and it has been suggested that the deregulated production of adipokines, such as leptin and adiponectin, are involved in IBD pathogenesis [63,64]. 

Our data reflect the knowledge that leptin downregulates PPAR-γ expression on the one hand, while inducing PI3K/mTOR signaling on the other. In turn, these conditions lead to the secretion of proinflammatory cytokines that initiate and maintain the inflammatory process. 

Our data suggest that the abuse of a HFD w/Suc directly impairs the homeostasis of enterocytes and the integrity of the intestinal mucosa barrier. The local accumulation of lipid droplets can certainly interfere with the physiological functions of the intestinal mucosa. Currently, a crucial point is to clarify whether these alterations can be reversible or even promote a local chronic inflammation, as occurs in NASH. It could be hypothesized that a HFD w/Suc could cause not only hepatic steatosis but also an “intestinal steatosis”. 

Another important feature will be to verify whether the morphological and inflammatory intestinal changes are only related to the intake of lipids or whether they are also induced and maintained by specific alterations of the gut microbiome determined by the hyperlipidic diet [65,66]. The gut microbiome is a complex and dynamic system, which is crucial for the development and maturation of both systemic and intestinal immune responses. The complex interaction between specific nutrients, the microbiome, and the immune system are central regulators in maintaining the intestinal homeostasis. Increasing evidence indicates that the gut microbiome and modifications induced by the consumption of a diet high in saturated fats and low in fibers, can trigger factors regulating the development and/or progression of chronic intestinal inflammation [65,66,67,68,69,70].

If confirmed by other studies, the metabolic and molecular alterations, as well as the alterations of the gut microbiome, could represent new therapeutic targets for the prevention and treatment of intestinal inflammatory disorders.

## 4. Materials and Methods

### 4.1. Animals and Experimental Design

This study employed paraffin-embedded bowel specimens harvested from our previous experimental procedures carried out at the University of L’Aquila, Department of Biotechnological and Applied Clinical Sciences (L’Aquila, Italy). The project was approved by the Italian Ministry of Health and the internal Committee of the University of L’Aquila. All experiments complied with the governmental guidelines (European Economic Community Council Directive 86/609, OJ 358, 1 Dec 12, 1987; Italian Legislative Decree 116/92, Gazzetta Ufficiale della Repubblica Italiana n. 40, 18 February 1992; National Institutes of Health Guide for the Care and Use of Laboratory Animals, NIH publication no. 85-23, 1985). All efforts were made to minimize animal suffering.

Fifteen male C57BL/6 mice (Charles Rivers Laboratories, Lyon, France) were used in the study. All animals were housed in plastic cages and kept in a pathogen-free environment, at constant room temperature, with a 12 h/12 h light/dark cycle. We provided animals with 20 g dry weight of food, at 100 g animal/d, according to Animal Research Review Panel guidelines. The animals were housed two or three per cage to guarantee social behavior and equal access to food.

### 4.2. Administration of Different Diets 

At 20 days of life, the mice were randomly divided into two groups: standard diet (SD, *n* = 5; used as the control), HFD w/Suc (*n* = 10), and LF-HCD (*n* = 10).

The main difference between the diets was the fat content. In particular, the fat source in the HFD w/Suc (D12331; Research Diets) was derived from hydrogenated coconut oil, while the fat source in the SD (2018S; Teklad laboratory, Envigo) was represented by ether extract. The carbohydrate source in control LF-HCD was sucrose (D12329 Research Diets). The detailed description of diet components is reported in Table 1. All animals were monitored daily for fluid and food intake and for signs of suffering, including reduced mobility, a decrease of social interactions, and raised fur.

### 4.3. Sample Recovery and Preparation

All animals were sacrificed after 18 months of differentiated diets. Following laparotomy, the small intestine was identified and rapidly excised. The samples were then fixed in 4% buffered formaldehyde and embedded in paraffin for further evaluation.

### 4.4. Histological and Immunohistochemical Analysis

Small intestine fragments of all animals were washed and immediately placed in 4% buffered formalin in phosphate buffer saline (PBS) at pH 7.4 for 3 h at room temperature before being embedded in low-temperature fusion paraffin. Sections of 3 μm thickness were stained with hematoxylin and eosin (H&E) to highlight the degree of inflammation, Masson’s trichrome to detect the deposition of connective tissue and fibrosis, and periodic acid–Schiff (PAS) to assess changes in the amount of goblet cells.

The stained sections were then observed under an Olympus BX51 Light Microscope (Olympus Optical Co. Ltd., Tokyo, Japan).

For immunohistochemical analysis, the samples were incubated for 40 min in methanol and then in 3% hydrogen peroxide solution for 5 min. The specimens were incubated overnight at 4 °C with the specific antibodies reported in Table 2.

Finally, the specimens were counterstained with Mayer’s hematoxylin, mounted, and observed under an Olympus BX51 light microscope (Olympus, Optical Co. Ltd., Segrate, Italy). To control the specificity of the immunohistochemistry, all reactions included negative controls (sections were incubated omitting the primary antibody).

### 4.5. Semiquantitative Digital Image Analysis of Immunohistochemical Staining

Semi-quantitative comparison of immunohistochemical staining was measured using the ImageJ immunohistochemistry (IHC) profiler software plugin, a digital image analysis public domain software [71]. Immunopositivity was expressed as a percentage of the total software-classified areas, and the data obtained were plotted as histograms. 

### 4.6. Statistical Analyses

The student’s *t* test was used for statistical analyses. Results were expressed as mean ± standard deviation; a *p*-value < 0.05 was considered statistically significant.

## Figures and Tables

**Table 1:** Composition of diets used in the study.

Ingredients (g%)	SD Diet	HFD w/Suc Diet	LF-HC Diet
Protein	18.6	23	16.8
Carbohydrate:	44.2	35.5	74.3
- Maltodextrin	-	17	12.5
- Sucrose	-	17.5	61.2
Fat:	6.2	35.8	4.8
- Soybean oil	-	2.5	1.8
- Coconut oil	-	33.3	2.9

SD = standard diet; HFD w/Suc = high fat with sucrose diet; LF-HCD = low fat, high-carbohydrate diet.

**Table 2:** Antibodies used with their sources and dilutions.

Antibody	Source	Dilution
Perilipin 1	Santa Cruz Biotechnology Inc., Santa Cruz, CA, USA; code sc-390169	1:100
Leptin	OriGene Technologies Inc., Rockville, MD, USA; code TA321088	1:50
Leptin Receptor	Santa Cruz Biotechnology Inc., Santa Cruz, CA, USA; code sc-8325	1:50
Adiponectin	Boster Biological Technology, Pleasanton, CA, USA; code PA2014-1	1:50
Adiponectin Receptor	Santa Cruz Biotechnology Inc., Santa Cruz, CA, USA; code sc-46748	1:50
PPAR-γ	Santa Cruz Biotechnology Inc., Santa Cruz, CA, USA; code sc-7273	1:100
p-mTOR	Santa Cruz Biotechnology Inc., Santa Cruz, CA, USA; code sc-293133	1:100
PI3K	Thermo-Fisher, Waltham, Massachusetts, USA; code PA5-28070	1:100
p-Akt	Biorbyt Ltd., Orwell Furlong, Cowley Rd, Cambridge, UK; code orb397210	1:100
TNF-α	Biorbyt Ltd., Orwell Furlong, Cowley Rd, Cambridge, UK; code orb323199	1:100
Zonulin 1	Cell signaling Technology, Inc. Danvers, MA, USA; code D6L1E	1:100
